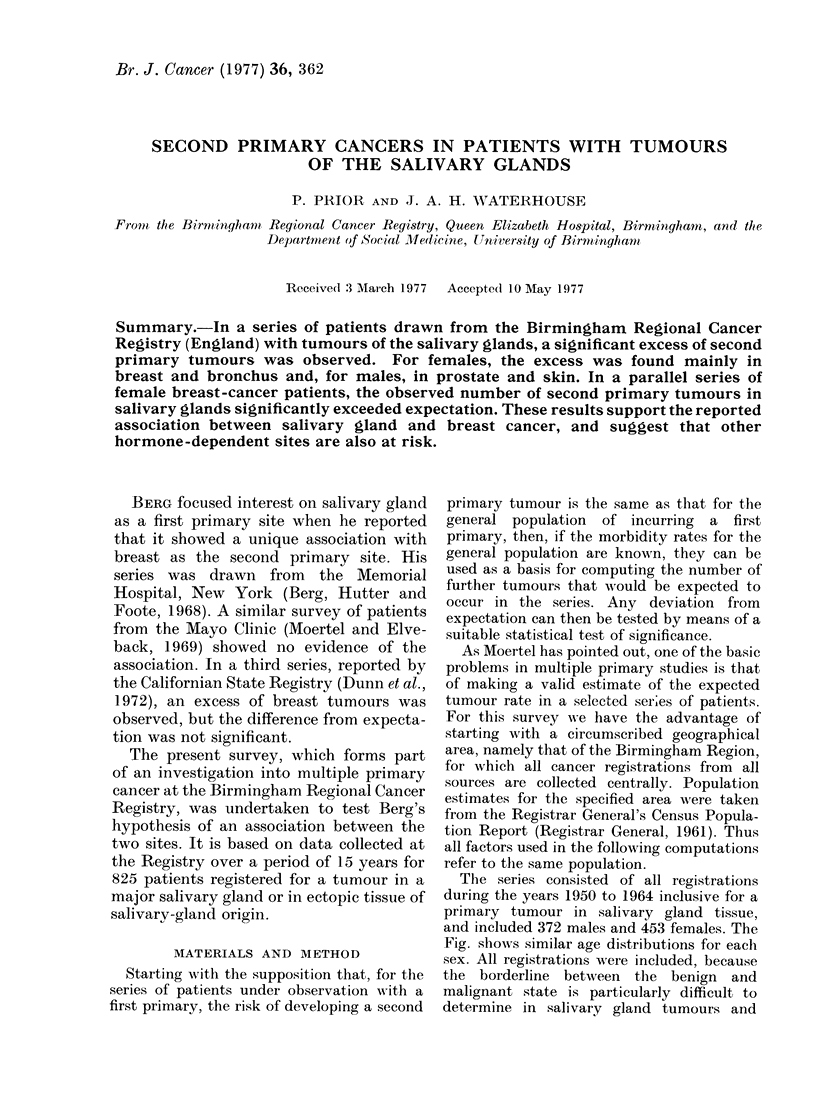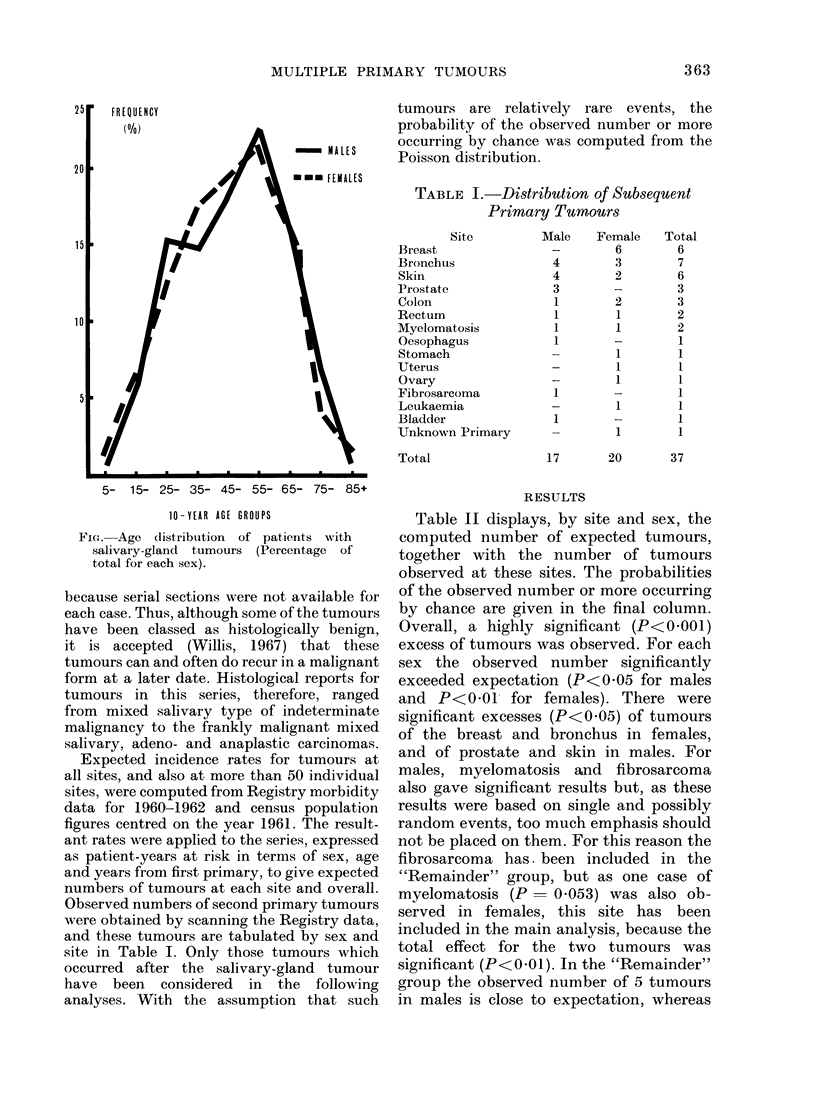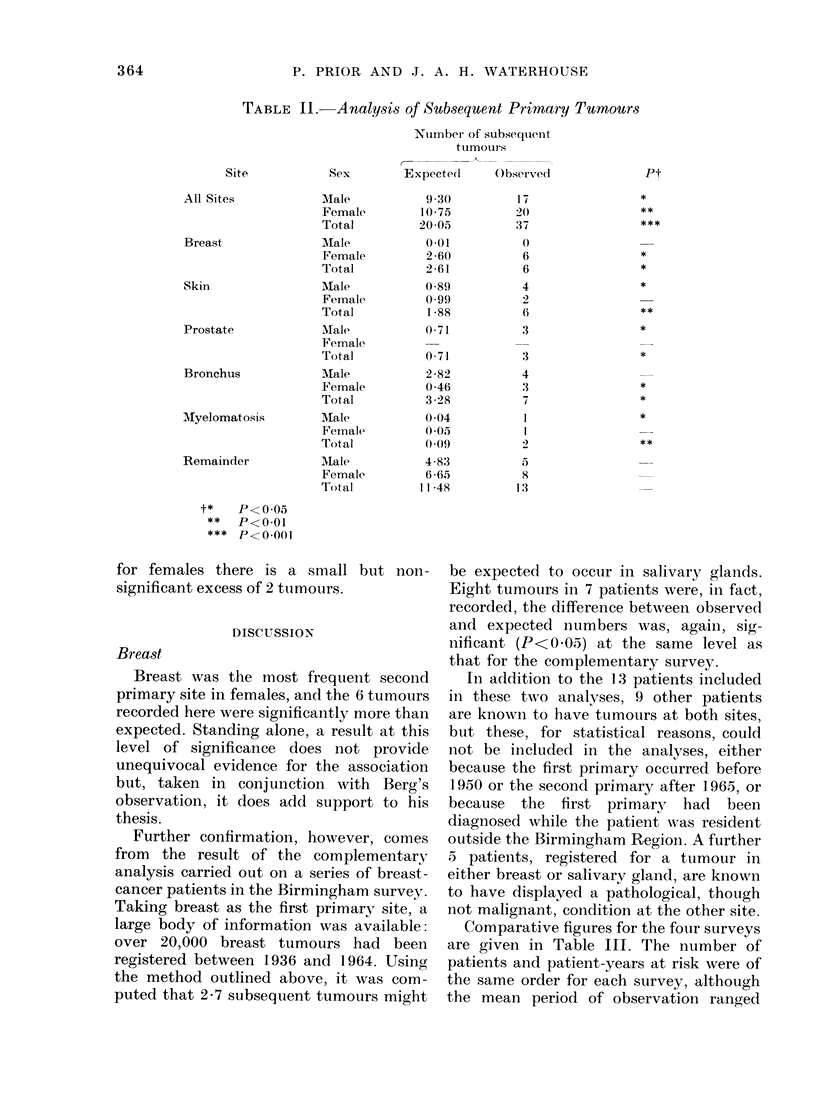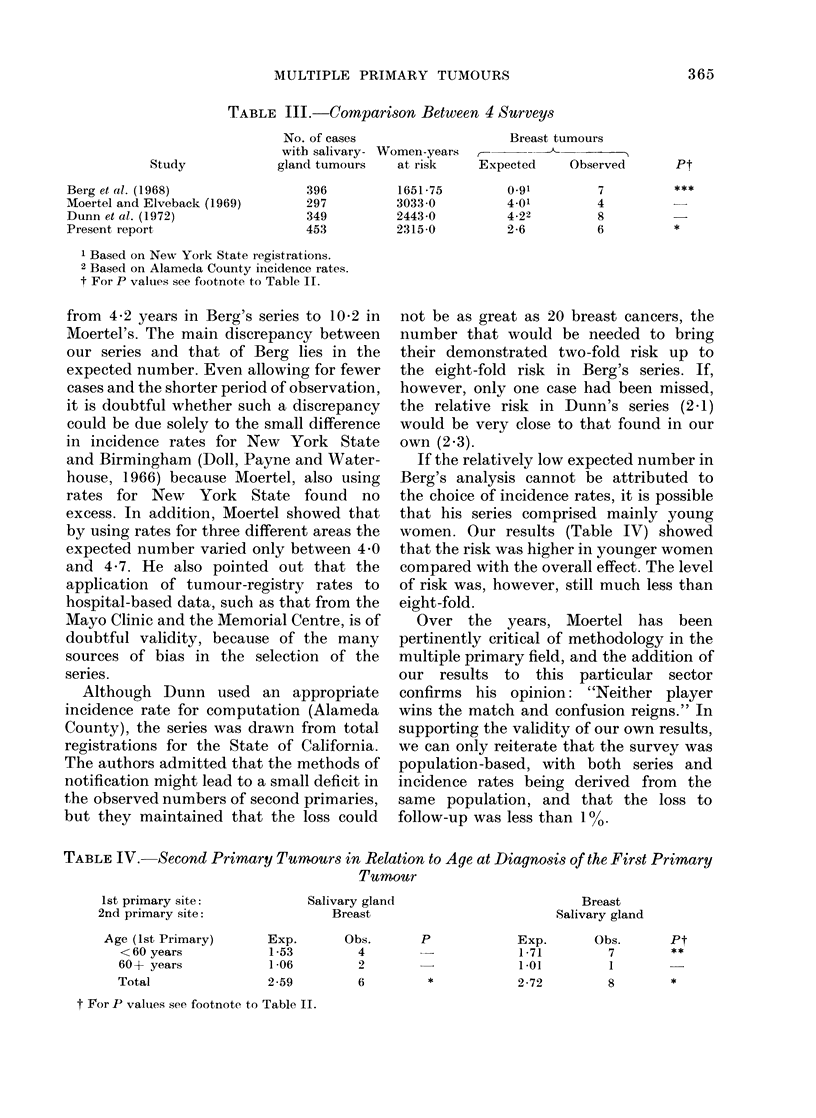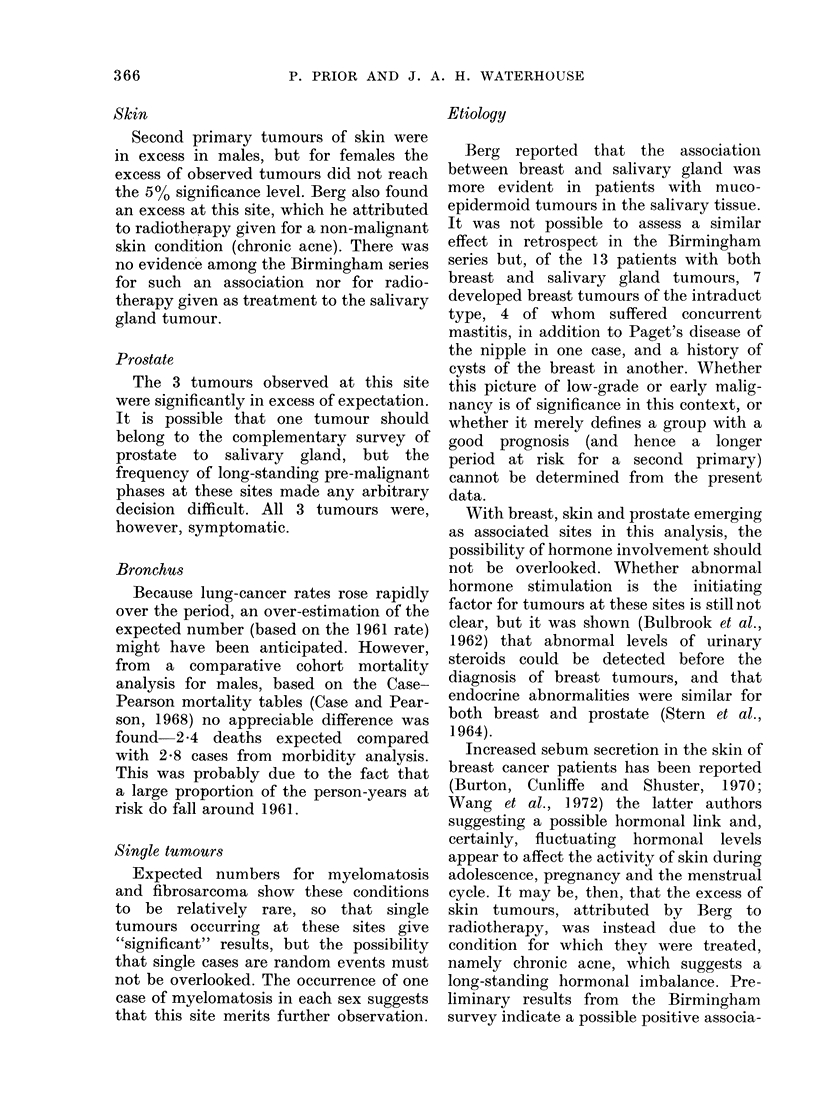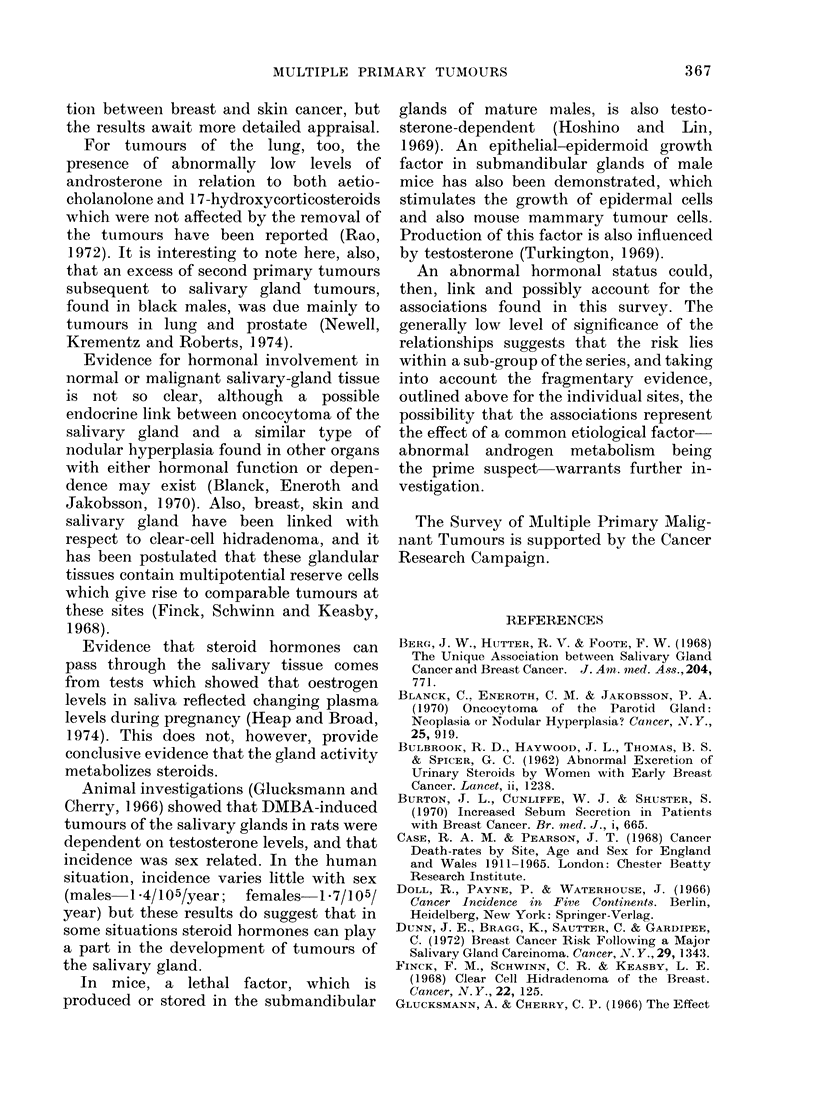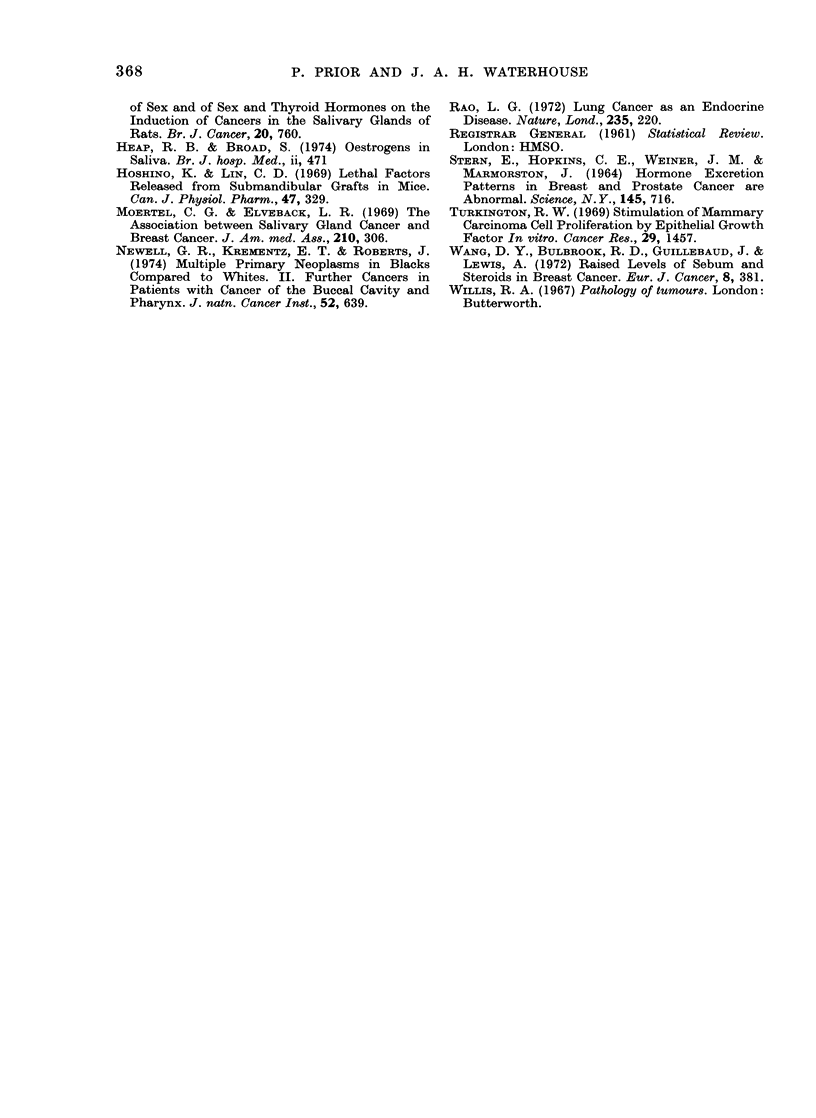# Second primary cancers in patients with tumours of the salivary glands.

**DOI:** 10.1038/bjc.1977.201

**Published:** 1977-09

**Authors:** P. Prior, J. A. Waterhouse

## Abstract

In a series of patients drawn from the Birmingham Regional Cancer Registry (England) with tumours of the salivary glands, a significant excess of second primary tumours was observed. For females, the excess was found mainly in breast and bronchus and, for males, in prostate and skin. In a parallel series of female breast-cancer patients, the observed number of second primary tumours in salivary glands significantly exceeded expectation. These results support the reported association between salivary gland and breast cancer, and suggest that other hormone-dependent sites are also at risk.


*Br. J. Cancer* (1977) **36**, 362

### SECOND PRIMARY CANCERS IN PATIENTS WITH TUMOURS OF THE SALIVARY GLANDS

P. PRIOR AND J. A. H. WATERHOUSE

*From the Birmingham Regional Cancer Registry, Queen Elizabeth Hospital, Birmingham, and the Department of Social Medicine, University of Birmingham*

Received 3 March 1977 Accepted 10 May 1977

**Summary.—In a series of patients drawn from the Birmingham Regional Cancer Registry (England) with tumours of the salivary glands, a significant excess of second primary tumours was observed. For females, the excess was found mainly in breast and bronchus and, for males, in prostate and skin. In a parallel series of female breast-cancer patients, the observed number of second primary tumours in salivary glands significantly exceeded expectation. These results support the reported association between salivary gland and breast cancer, and suggest that other hormone-dependent sites are also at risk.**

BERG focused interest on salivary gland as a first primary site when he reported that it showed a unique association with breast as the second primary site. His series was drawn from the Memorial Hospital, New York (Berg, Hutter and Foote, 1968). A similar survey of patients from the Mayo Clinic (Moertel and Elveback, 1969) showed no evidence of the association. In a third series, reported by the Californian State Registry (Dunn *et al.*, 1972), an excess of breast tumours was observed, but the difference from expectation was not significant.

The present survey, which forms part of an investigation into multiple primary cancer at the Birmingham Regional Cancer Registry, was undertaken to test Berg's hypothesis of an association between the two sites. It is based on data collected at the Registry over a period of 15 years for 825 patients registered for a tumour in a major salivary gland or in ectopic tissue of salivary-gland origin.

### MATERIALS AND METHOD

Starting with the supposition that, for the series of patients under observation with a first primary, the risk of developing a second primary tumour is the same as that for the general population of incurring a first primary, then, if the morbidity rates for the general population are known, they can be used as a basis for computing the number of further tumours that would be expected to occur in the series. Any deviation from expectation can then be tested by means of a suitable statistical test of significance.

As Moertel has pointed out, one of the basic problems in multiple primary studies is that of making a valid estimate of the expected tumour rate in a selected series of patients. For this survey we have the advantage of starting with a circumscribed geographical area, namely that of the Birmingham Region, for which all cancer registrations from all sources are collected centrally. Population estimates for the specified area were taken from the Registrar General's Census Population Report (Registrar General, 1961). Thus all factors used in the following computations refer to the same population.

The series consisted of all registrations during the years 1950 to 1964 inclusive for a primary tumour in salivary gland tissue, and included 372 males and 453 females. The Fig. shows similar age distributions for each sex. All registrations were included, because the borderline between the benign and malignant state is particularly difficult to determine in salivary gland tumours and

FIG.—Age distribution of patients with salivary-gland tumours (Percentage of total for each sex).

because serial sections were not available for each case. Thus, although some of the tumours have been classed as histologically benign, it is accepted (Willis, 1967) that these tumours can and often do recur in a malignant form at a later date. Histological reports for tumours in this series, therefore, ranged from mixed salivary type of indeterminate malignancy to the frankly malignant mixed salivary, adeno- and anaplastic carcinomas.

Expected incidence rates for tumours at all sites, and also at more than 50 individual sites, were computed from Registry morbidity data for 1960–1962 and census population figures centred on the year 1961. The resultant rates were applied to the series, expressed as patient-years at risk in terms of sex, age and years from first primary, to give expected numbers of tumours at each site and overall. Observed numbers of second primary tumours were obtained by scanning the Registry data, and these tumours are tabulated by sex and site in Table I. Only those tumours which occurred after the salivary-gland tumour have been considered in the following analyses. With the assumption that such tumours are relatively rare events, the probability of the observed number or more occurring by chance was computed from the Poisson distribution.

TABLE I.—*Distribution of Subsequent Primary Tumours*

| Site | Male | Female | Total |
|---|---|---|---|
| Breast | – | 6 | 6 |
| Bronchus | 4 | 3 | 7 |
| Skin | 4 | 2 | 6 |
| Prostate | 3 | – | 3 |
| Colon | 1 | 2 | 3 |
| Rectum | 1 | 1 | 2 |
| Myelomatosis | 1 | 1 | 2 |
| Oesophagus | 1 | – | 1 |
| Stomach | – | 1 | 1 |
| Uterus | – | 1 | 1 |
| Ovary | – | 1 | 1 |
| Fibrosarcoma | 1 | – | 1 |
| Leukaemia | – | 1 | 1 |
| Bladder | 1 | – | 1 |
| Unknown Primary | – | 1 | 1 |
| Total | 17 | 20 | 37 |

RESULTS

Table II displays, by site and sex, the computed number of expected tumours, together with the number of tumours observed at these sites. The probabilities of the observed number or more occurring by chance are given in the final column. Overall, a highly significant ($P<0{\cdot}001$) excess of tumours was observed. For each sex the observed number significantly exceeded expectation ($P<0{\cdot}05$ for males and $P<0{\cdot}01$ for females). There were significant excesses ($P<0{\cdot}05$) of tumours of the breast and bronchus in females, and of prostate and skin in males. For males, myelomatosis and fibrosarcoma also gave significant results but, as these results were based on single and possibly random events, too much emphasis should not be placed on them. For this reason the fibrosarcoma has been included in the "Remainder" group, but as one case of myelomatosis ($P = 0{\cdot}053$) was also observed in females, this site has been included in the main analysis, because the total effect for the two tumours was significant ($P<0{\cdot}01$). In the "Remainder" group the observed number of 5 tumours in males is close to expectation, whereas

TABLE II.—*Analysis of Subsequent Primary Tumours*

| Site | Sex | Number of subsequent tumours: Expected | Observed | P† |
|---|---|---|---|---|
| All Sites | Male | 9·30 | 17 | * |
| | Female | 10·75 | 20 | ** |
| | Total | 20·05 | 37 | *** |
| Breast | Male | 0·01 | 0 | — |
| | Female | 2·60 | 6 | * |
| | Total | 2·61 | 6 | * |
| Skin | Male | 0·89 | 4 | * |
| | Female | 0·99 | 2 | — |
| | Total | 1·88 | 6 | ** |
| Prostate | Male | 0·71 | 3 | * |
| | Female | — | — | — |
| | Total | 0·71 | 3 | * |
| Bronchus | Male | 2·82 | 4 | — |
| | Female | 0·46 | 3 | * |
| | Total | 3·28 | 7 | * |
| Myelomatosis | Male | 0·04 | 1 | * |
| | Female | 0·05 | 1 | — |
| | Total | 0·09 | 2 | ** |
| Remainder | Male | 4·83 | 5 | — |
| | Female | 6·65 | 8 | — |
| | Total | 11·48 | 13 | — |

†* $P<0·05$
** $P<0·01$
*** $P<0·001$

for females there is a small but non-significant excess of 2 tumours.

### DISCUSSION

### *Breast*

Breast was the most frequent second primary site in females, and the 6 tumours recorded here were significantly more than expected. Standing alone, a result at this level of significance does not provide unequivocal evidence for the association but, taken in conjunction with Berg's observation, it does add support to his thesis.

Further confirmation, however, comes from the result of the complementary analysis carried out on a series of breast-cancer patients in the Birmingham survey. Taking breast as the first primary site, a large body of information was available: over 20,000 breast tumours had been registered between 1936 and 1964. Using the method outlined above, it was computed that 2·7 subsequent tumours might be expected to occur in salivary glands. Eight tumours in 7 patients were, in fact, recorded, the difference between observed and expected numbers was, again, significant ($P<0·05$) at the same level as that for the complementary survey.

In addition to the 13 patients included in these two analyses, 9 other patients are known to have tumours at both sites, but these, for statistical reasons, could not be included in the analyses, either because the first primary occurred before 1950 or the second primary after 1965, or because the first primary had been diagnosed while the patient was resident outside the Birmingham Region. A further 5 patients, registered for a tumour in either breast or salivary gland, are known to have displayed a pathological, though not malignant, condition at the other site.

Comparative figures for the four surveys are given in Table III. The number of patients and patient-years at risk were of the same order for each survey, although the mean period of observation ranged

TABLE III.—*Comparison Between 4 Surveys*

| Study | No. of cases with salivary-gland tumours | Women-years at risk | Breast tumours Expected | Breast tumours Observed | P† |
|---|---|---|---|---|---|
| Berg *et al.* (1968) | 396 | 1651·75 | 0·9[1] | 7 | *** |
| Moertel and Elveback (1969) | 297 | 3033·0 | 4·0[1] | 4 | — |
| Dunn *et al.* (1972) | 349 | 2443·0 | 4·2[2] | 8 | — |
| Present report | 453 | 2315·0 | 2·6 | 6 | * |

[1] Based on New York State registrations.
[2] Based on Alameda County incidence rates.
† For *P* values see footnote to Table II.

from 4·2 years in Berg's series to 10·2 in Moertel's. The main discrepancy between our series and that of Berg lies in the expected number. Even allowing for fewer cases and the shorter period of observation, it is doubtful whether such a discrepancy could be due solely to the small difference in incidence rates for New York State and Birmingham (Doll, Payne and Waterhouse, 1966) because Moertel, also using rates for New York State found no excess. In addition, Moertel showed that by using rates for three different areas the expected number varied only between 4·0 and 4·7. He also pointed out that the application of tumour-registry rates to hospital-based data, such as that from the Mayo Clinic and the Memorial Centre, is of doubtful validity, because of the many sources of bias in the selection of the series.

Although Dunn used an appropriate incidence rate for computation (Alameda County), the series was drawn from total registrations for the State of California. The authors admitted that the methods of notification might lead to a small deficit in the observed numbers of second primaries, but they maintained that the loss could not be as great as 20 breast cancers, the number that would be needed to bring their demonstrated two-fold risk up to the eight-fold risk in Berg's series. If, however, only one case had been missed, the relative risk in Dunn's series (2·1) would be very close to that found in our own (2·3).

If the relatively low expected number in Berg's analysis cannot be attributed to the choice of incidence rates, it is possible that his series comprised mainly young women. Our results (Table IV) showed that the risk was higher in younger women compared with the overall effect. The level of risk was, however, still much less than eight-fold.

Over the years, Moertel has been pertinently critical of methodology in the multiple primary field, and the addition of our results to this particular sector confirms his opinion: "Neither player wins the match and confusion reigns." In supporting the validity of our own results, we can only reiterate that the survey was population-based, with both series and incidence rates being derived from the same population, and that the loss to follow-up was less than 1%.

TABLE IV.—*Second Primary Tumours in Relation to Age at Diagnosis of the First Primary Tumour*

| 1st primary site: / 2nd primary site: | Salivary gland / Breast | | | Breast / Salivary gland | | |
|---|---|---|---|---|---|---|
| Age (1st Primary) | Exp. | Obs. | *P* | Exp. | Obs. | *P*† |
| <60 years | 1·53 | 4 | — | 1·71 | 7 | ** |
| 60+ years | 1·06 | 2 | — | 1·01 | 1 | — |
| Total | 2·59 | 6 | * | 2·72 | 8 | * |

† For *P* values see footnote to Table II.

*Skin*

Second primary tumours of skin were in excess in males, but for females the excess of observed tumours did not reach the 5% significance level. Berg also found an excess at this site, which he attributed to radiotherapy given for a non-malignant skin condition (chronic acne). There was no evidence among the Birmingham series for such an association nor for radiotherapy given as treatment to the salivary gland tumour.

*Prostate*

The 3 tumours observed at this site were significantly in excess of expectation. It is possible that one tumour should belong to the complementary survey of prostate to salivary gland, but the frequency of long-standing pre-malignant phases at these sites made any arbitrary decision difficult. All 3 tumours were, however, symptomatic.

*Bronchus*

Because lung-cancer rates rose rapidly over the period, an over-estimation of the expected number (based on the 1961 rate) might have been anticipated. However, from a comparative cohort mortality analysis for males, based on the Case–Pearson mortality tables (Case and Pearson, 1968) no appreciable difference was found—2·4 deaths expected compared with 2·8 cases from morbidity analysis. This was probably due to the fact that a large proportion of the person-years at risk do fall around 1961.

*Single tumours*

Expected numbers for myelomatosis and fibrosarcoma show these conditions to be relatively rare, so that single tumours occurring at these sites give "significant" results, but the possibility that single cases are random events must not be overlooked. The occurrence of one case of myelomatosis in each sex suggests that this site merits further observation.

*Etiology*

Berg reported that the association between breast and salivary gland was more evident in patients with muco-epidermoid tumours in the salivary tissue. It was not possible to assess a similar effect in retrospect in the Birmingham series but, of the 13 patients with both breast and salivary gland tumours, 7 developed breast tumours of the intraduct type, 4 of whom suffered concurrent mastitis, in addition to Paget's disease of the nipple in one case, and a history of cysts of the breast in another. Whether this picture of low-grade or early malignancy is of significance in this context, or whether it merely defines a group with a good prognosis (and hence a longer period at risk for a second primary) cannot be determined from the present data.

With breast, skin and prostate emerging as associated sites in this analysis, the possibility of hormone involvement should not be overlooked. Whether abnormal hormone stimulation is the initiating factor for tumours at these sites is still not clear, but it was shown (Bulbrook *et al.*, 1962) that abnormal levels of urinary steroids could be detected before the diagnosis of breast tumours, and that endocrine abnormalities were similar for both breast and prostate (Stern *et al.*, 1964).

Increased sebum secretion in the skin of breast cancer patients has been reported (Burton, Cunliffe and Shuster, 1970; Wang *et al.*, 1972) the latter authors suggesting a possible hormonal link and, certainly, fluctuating hormonal levels appear to affect the activity of skin during adolescence, pregnancy and the menstrual cycle. It may be, then, that the excess of skin tumours, attributed by Berg to radiotherapy, was instead due to the condition for which they were treated, namely chronic acne, which suggests a long-standing hormonal imbalance. Preliminary results from the Birmingham survey indicate a possible positive association between breast and skin cancer, but the results await more detailed appraisal.

For tumours of the lung, too, the presence of abnormally low levels of androsterone in relation to both aetiocholanolone and 17-hydroxycorticosteroids which were not affected by the removal of the tumours have been reported (Rao, 1972). It is interesting to note here, also, that an excess of second primary tumours subsequent to salivary gland tumours, found in black males, was due mainly to tumours in lung and prostate (Newell, Krementz and Roberts, 1974).

Evidence for hormonal involvement in normal or malignant salivary-gland tissue is not so clear, although a possible endocrine link between oncocytoma of the salivary gland and a similar type of nodular hyperplasia found in other organs with either hormonal function or dependence may exist (Blanck, Eneroth and Jakobsson, 1970). Also, breast, skin and salivary gland have been linked with respect to clear-cell hidradenoma, and it has been postulated that these glandular tissues contain multipotential reserve cells which give rise to comparable tumours at these sites (Finck, Schwinn and Keasby, 1968).

Evidence that steroid hormones can pass through the salivary tissue comes from tests which showed that oestrogen levels in saliva reflected changing plasma levels during pregnancy (Heap and Broad, 1974). This does not, however, provide conclusive evidence that the gland activity metabolizes steroids.

Animal investigations (Glucksmann and Cherry, 1966) showed that DMBA-induced tumours of the salivary glands in rats were dependent on testosterone levels, and that incidence was sex related. In the human situation, incidence varies little with sex (males—$1{\cdot}4/10^5$/year; females—$1{\cdot}7/10^5$/year) but these results do suggest that in some situations steroid hormones can play a part in the development of tumours of the salivary gland.

In mice, a lethal factor, which is produced or stored in the submandibular glands of mature males, is also testosterone-dependent (Hoshino and Lin, 1969). An epithelial–epidermoid growth factor in submandibular glands of male mice has also been demonstrated, which stimulates the growth of epidermal cells and also mouse mammary tumour cells. Production of this factor is also influenced by testosterone (Turkington, 1969).

An abnormal hormonal status could, then, link and possibly account for the associations found in this survey. The generally low level of significance of the relationships suggests that the risk lies within a sub-group of the series, and taking into account the fragmentary evidence, outlined above for the individual sites, the possibility that the associations represent the effect of a common etiological factor—abnormal androgen metabolism being the prime suspect—warrants further investigation.

The Survey of Multiple Primary Malignant Tumours is supported by the Cancer Research Campaign.

### REFERENCES

Berg, J. W., Hutter, R. V. & Foote, F. W. (1968) The Unique Association between Salivary Gland Cancer and Breast Cancer. *J. Am. med. Ass.*, **204**, 771.

Blanck, C., Eneroth, C. M. & Jakobsson, P. A. (1970) Oncocytoma of the Parotid Gland: Neoplasia or Nodular Hyperplasia? *Cancer, N.Y.*, **25**, 919.

Bulbrook, R. D., Haywood, J. L., Thomas, B. S. & Spicer, G. C. (1962) Abnormal Excretion of Urinary Steroids by Women with Early Breast Cancer. *Lancet*, ii, 1238.

Burton, J. L., Cunliffe, W. J. & Shuster, S. (1970) Increased Sebum Secretion in Patients with Breast Cancer. *Br. med. J.*, i, 665.

Case, R. A. M. & Pearson, J. T. (1968) Cancer Death-rates by Site, Age and Sex for England and Wales 1911–1965. London: Chester Beatty Research Institute.

Doll, R., Payne, P. & Waterhouse, J. (1966) *Cancer Incidence in Five Continents*. Berlin, Heidelberg, New York: Springer-Verlag.

Dunn, J. E., Bragg, K., Sautter, C. & Gardipee, C. (1972) Breast Cancer Risk Following a Major Salivary Gland Carcinoma. *Cancer, N.Y.*, **29**, 1343.

Finck, F. M., Schwinn, C. R. & Keasby, L. E. (1968) Clear Cell Hidradenoma of the Breast. *Cancer, N.Y.*, **22**, 125.

Glucksmann, A. & Cherry, C. P. (1966) The Effect of Sex and of Sex and Thyroid Hormones on the Induction of Cancers in the Salivary Glands of Rats. *Br. J. Cancer*, **20**, 760.

HEAP, R. B. & BROAD, S. (1974) Oestrogens in Saliva. *Br. J. hosp. Med.*, ii, 471

HOSHINO, K. & LIN, C. D. (1969) Lethal Factors Released from Submandibular Grafts in Mice. *Can. J. Physiol. Pharm.*, **47**, 329.

MOERTEL, C. G. & ELVEBACK, L. R. (1969) The Association between Salivary Gland Cancer and Breast Cancer. *J. Am. med. Ass.*, **210**, 306.

NEWELL, G. R., KREMENTZ, E. T. & ROBERTS, J. (1974) Multiple Primary Neoplasms in Blacks Compared to Whites. II. Further Cancers in Patients with Cancer of the Buccal Cavity and Pharynx. *J. natn. Cancer Inst.*, **52**, 639.

RAO, L. G. (1972) Lung Cancer as an Endocrine Disease. *Nature, Lond.*, **235**, 220.

REGISTRAR GENERAL (1961) *Statistical Review.* London: HMSO.

STERN, E., HOPKINS, C. E., WEINER, J. M. & MARMORSTON, J. (1964) Hormone Excretion Patterns in Breast and Prostate Cancer are Abnormal. *Science, N.Y.*, **145**, 716.

TURKINGTON, R. W. (1969) Stimulation of Mammary Carcinoma Cell Proliferation by Epithelial Growth Factor *In vitro. Cancer Res.*, **29**, 1457.

WANG, D. Y., BULBROOK, R. D., GUILLEBAUD, J. & LEWIS, A. (1972) Raised Levels of Sebum and Steroids in Breast Cancer. *Eur. J. Cancer*, **8**, 381.

WILLIS, R. A. (1967) *Pathology of tumours.* London: Butterworth.